# Immunogenicity of poliovirus vaccines in chronically malnourished infants: A randomized controlled trial in Pakistan

**DOI:** 10.1016/j.vaccine.2015.04.055

**Published:** 2015-06-04

**Authors:** Ali Faisal Saleem, Ondrej Mach, Farheen Quadri, Asia Khan, Zaid Bhatti, Najeeb ur Rehman, Sohail Zaidi, William C. Weldon, Steven M. Oberste, Maha Salama, Roland W. Sutter, Anita K.M. Zaidi

**Affiliations:** a Department of Pediatrics and Child Health, Aga Khan University, Karachi, Pakistan; b Polio Eradication Department, World Health Organization, Geneva, Switzerland; c Department of virology, National Institute of Health, Islamabad, Pakistan; d Polio and Picornavirus Laboratory Branch, Centers for Disease Control and Prevention, Atlanta, USA; e Population Immunity Laboratory, Polio and Picornavirus Laboratory Branch, Centers for Disease Control and Prevention, Atlanta, USA; f VACSERA Holding Company, Cairo, Egypt

**Keywords:** Chronic malnutrition, Polio vaccine immunity, Seroconversion, Polio vaccine trial

## Abstract

Reaching high population immunity against polioviruses (PV) is essential to achieving global polio eradication. Efficacy of oral poliovirus vaccine (OPV) varies and is lower among children living in tropical areas with impoverished environments. Malnutrition found as a risk factor for lower serological protection against PV. We compared whether inactivated polio vaccine (IPV) can be used to rapidly close the immunity gap among chronically malnourished (stunted) infants in Pakistan who will not be eligible for the 14 week IPV dose in routine EPI schedule. A phase 3, multicenter 4-arm randomized controlled trial conducted at five Primary Health Care (PHC) centers in Karachi, Pakistan. Infants, 9–12 months were stratified by length for age Z score into chronically malnourished and normally nourished. Infants were randomized to receive one dose of either bivalent OPV (bOPV) alone or bOPV + IPV. Baseline seroprevalence of PV antibodies and serum immune response to study vaccine dose were assessed by neutralization assay. Vaccine PV shedding in stool was evaluated 7 days after a bOPV challenge dose. Sera and stool were analyzed from 852/928 (92%) enrolled children. At baseline, the seroprevalence was 85.6% (*n* = 386), 73.6% (*n* = 332), and 70.7% (*n* = 319) in malnourished children against PV types 1, 2 and 3 respectively; and 94.1% (*n* = 448), 87.0% (*n* = 441) and 83.6% (*n* = 397) in the normally nourished group (*p* < 0.05). Children had previously received 9–10 doses of bOPV (80%) or tOPV (20%). One dose of IPV + bOPV given to malnourished children increased their serological protection (PV1, *n* = 201, 97.6%; PV2, *n* = 198, 96.1% and PV3, *n* = 189, 91.7%) to parity with normally nourished children who had not received IPV (*p* = <0.001). Seroconversion and boosting for all three serotypes was significantly more frequent in children who received IPV + bOPV than in those with bOPV only (*p* < 0.001) in both strata. Shedding of polioviruses in stool did not differ between study groups and ranged from 2.4% (*n* = 5) to 7.1% (*n* = 15). In malnourished children the shedding was reduced after bOPV + IPV compared to bOPV only.

Chronically malnourished infants were more likely to be unprotected against polioviruses than normal infants. bOPV + IPV helped close the immunity gap better than bOPV alone.

## 1 Introduction

The goal of global eradication of poliomyelitis was adopted in 1988 and since then the number of paralyzed persons due to polioviruses has decreased by over 99.9%. In 2013, the World Health Organization (WHO) reported 416 cases of paralytic poliomyelitis due to wild polioviruses worldwide [1]. In mid-2014, the remaining endemic areas with wild poliovirus circulation were limited to security compromised parts of Pakistan, Afghanistan and Nigeria, however, exportations of wild polioviruses from these endemic areas into polio-free countries have occurred in multiple occasions, sometimes causing large outbreaks of poliomyelitis [2]. Thus, wild poliovirus exportations from the last endemic foci remain a constant threat to polio eradication.

Oral poliovirus vaccine (OPV) has been used in routine immunization and in supplementary immunization activities (SIAs) throughout the polio eradication initiative, and the dramatic decrease of poliomyelitis incidence is a result of massive OPV use [3]. In industrialized countries, the immunogenicity of OPV was considered adequate with seroconversion rates of approximately 80% or higher to any virus with a single monovalent OPV dose [4]. In these settings, three doses of OPV were sufficient to provide close to 100% protection to all three poliovirus serotypes. However, in some developing countries the OPV immunogenicity was considerably lower with the mean frequency of response to any poliovirus serotype of 37–40% in South India and <20% in Northern India [5–7]. The reasons for the variation in immune response are likely multi-factorial; i.e., areas with high population density and poor standards of hygiene and sanitation appear to have lower OPV efficacy [6,8]. Several hypotheses attempted to explain this variation including diarrhea, concurrent intestinal infections, low zinc levels, low Vitamin A levels, and malnutrition [9,10]. Limited evidence suggests that repeated intestinal infections can lead to malnutrition and can reduce immunogenicity of other oral vaccines (e.g., rotavirus vaccine, typhoid vaccine) [11,12].

In Pakistan, the rates of chronic malnutrition (stunting) are high and documented to be about 43.7% among children under 5 years of age in a nationwide survey conducted in 2011, and 34.4% in 9–11 months old children [13]. In Pakistan between 2011 and 2013, the majority of cases of paralytic poliomyelitis were observed in vaccinated children coming from areas with elevated levels of chronic malnutrition [14].

OPV used in poliovirus eradication is either trivalent (tOPV) providing protection against all three poliovirus serotypes; bivalent OPV (bOPV) providing protection against serotypes 1 and 3; or monovalent OPV (mOPV1) providing protection against poliovirus type 1. The selection of OPV type for SIAs is driven by epidemiology and vaccine availability. All of these vaccines have been used in SIAs conducted in Pakistan.

The Polio Eradication and Endgame Strategic Plan 2013–2018 recommends that one dose of Inactivated Poliovirus Vaccine (IPV) is added to the routine immunization programs in all countries that currently use only OPV by the end of 2015 [15]. IPV has been successfully used in many polio-free countries to maintain population protection against polioviruses, and it has been demonstrated that IPV induces humoral immunity in naive children and boosts mucosal immunity in those who had previously received OPV [16–18]. IPV together with OPV has recently been used in SIAs to control outbreaks (Kenya) or to accelerate poliovirus eradication in persistent endemic areas (Nigeria) [19].

In this study we assessed the vaccine-induced serological and mucosal protection against poliovirus in malnourished and normal infants; and compared the immune responses between IPV + bOPV versus bOPV alone in malnourished and normal infants. The evaluation of differential immune response to poliovirus vaccines between malnourished and normal infants could lead to new strategies for polio eradication in areas with known high malnutrition rates.

## 2 Methods

This was a multicenter randomized controlled trial conducted at five Primary Health Care (PHC) centers in Karachi, Pakistan, between October 2012 and November 2013. The study area consisted of five low-income communities in and around Karachi (including contiguous coastal villages at the outskirts of Karachi, and one urban squatter settlement). The participating PHC centers are operated by the Department of Pediatrics and Child Health Research Program of the Aga Khan University and provide free primary health care services to children from these communities.

Parents of Infants 9–12 months of age were approached at home by health center staff, informed about the trial and invited to participate in the study. Inclusion criteria were infants 9–12 months old who have resided in the study area for the last 3 months. Children who were already enrolled in another polio study, who were acutely ill or required urgent medical care were excluded. Children suffering from acute malnutrition, defined by a low weight for height *z*-score (below −2 SD of the median WHO growth standards), or those who refused blood testing were also excluded. Further, children who had received a supplementary dose of OPV within the last four weeks before the trial start were excluded from the trial.

At the time of enrollment, the anthropometric measurements were taken to assess the nutritional status. The infants were divided into two groups: normally nourished and chronically malnourished (defined as height for age z-score below −2 SD of the median WHO growth standards). Both malnourished and normal children were randomized into one of two study arms providing a total of four study arms: (1) MAL A included chronically malnourished children randomized into bOPV only arm; (2) MAL B included chronically malnourished children randomized into bOPV + IPV arm; (3) NOR A included normally nourished children randomized into bOPV only arm; and (4) NOR B included normally nourished children randomized into bOPV + IPV arm (Table 1, demographic characteristics of study participants.). The study vaccine dose (bOPV only or bOPV + IPV) was administered at enrollment. Peripheral blood (minimum 1 mL) was collected at the time of enrollment (prior to the study vaccine administration) and after 28 days. A challenge dose of bOPV was administered to all study participants 28 days after enrollment. One stool sample was collected 28 days after enrollment (prior to bOPV challenge dose administration); and second stool sample was collected 7 days after the bOPV challenge dose administration.

Infants who provided both blood and stool samples, and did not receive other than the study OPV doses while enrolled, were considered to have completed the study “per protocol”.

OPV and IPV were obtained from WHO-prequalified producers: IPV from the Netherlands Vaccine Institute (NVI) and bOPV from GlaxoSmithKline. The bOPV was formulated to contain at least 10^6^ CCID_50_ of Sabin poliovirus type 1 and at least 10^5.8^ CCID_50_ of Sabin poliovirus type 3. Each IPV dose (0.5 mL) is formulated to contain 40 D antigen units of type 1, 8 D antigen units of type 2, and 32 D antigen units of type 3 poliovirus.

Blood specimens collected at the sites were allowed to clot, centrifuged to separate serum, and transported to the Infectious Disease Research Laboratory (IDRL) at the Aga Khan University where they were stored at -20 °C until shipment to the Centers for Disease Control and Prevention (CDC), Atlanta, Georgia, USA, where the sera were tested for presence of poliovirus neutralizing antibodies using standard neutralization assays [20].

Stool specimens were collected at the primary care clinic or at children's homes and stored at IDRL at +4 °C until shipment to WHO collaborating laboratory for Polio at the National Institute of Health in Islamabad, Pakistan, where it was tested for the presence of poliovirus using standard poliovirus detection methodology [21].

Seropositivity was defined as reciprocal titers of poliovirus neutralizing antibodies ≥8; seroconversion was defined as the change from seronegative to seropositive (from reciprocal titer of <8 to ≥8); and boosting was defined as ≥4-fold increase in titers. In this study, “immune response” combines both boosting and seroconversion. The analysis of immune response was restricted to infants with a baseline serological titer of ≤ 362 to ensure that a 4-fold boosting response could be achieved since the highest titer tested was 1:1,448. Shedding of poliovirus was defined as isolation of poliovirus in a stool sample.

Vaccination history with OPV received through routine immunization was assessed from vaccination cards when available and by parental recall. OPV doses received through SIAs were estimated by the number of SIA rounds that were conducted in the study area during the life of each child. Enrolled subjects were not vaccinated with any supplementary OPV doses through SIAs that were conducted during the study period.

Diarrhea was defined as three or more loose or watery stools per day. Parents were asked about episodes of diarrhea in the period of 7 days preceding enrolment.

Adverse events following vaccination were identified by site investigators and reviewed by the principal investigator. Children were observed for 30 min following the administration of the vaccine for immediate adverse events; parents were instructed to immediately report back to the health centers if adverse events occurred. Serious adverse events were reported for review by the Data and Safety Monitoring Board and by the Ethical Review Committees of the Aga Khan University and the World Health Organization.

A sample size of 190 evaluable infants in each study arm was estimated at 90% power and *α* set at 0.05 to detect differences of greater than 10% in seroconversion between the arms. Assuming a 10% attrition rate after randomization, the required sample size was 210 infants per study arm. Statistical analysis was performed using STATA version 12. The proportion of seroconversion in different study arms was compared by *χ*^2^ test for quantitative variables. Analysis of variance (ANOVA) was used to compare the mean difference across the study arms. *K*-sample equality of median test was performed to compare the median titers across the study arms and 95% confidence intervals for median titers were calculated.

## 3 Results

**Fig. 1:**
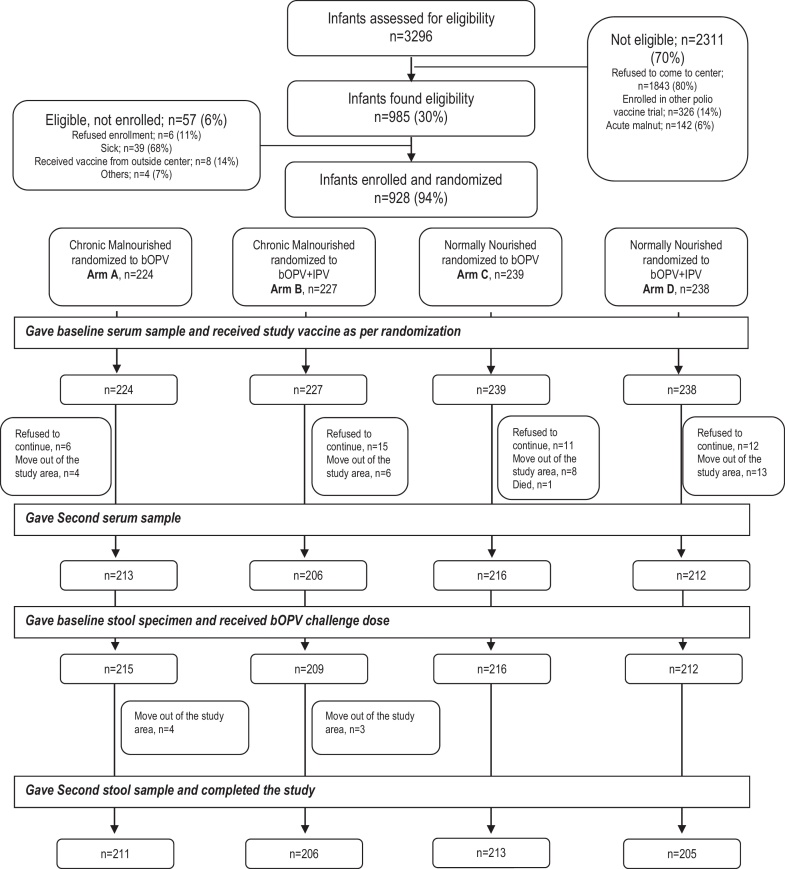
Trial Profile.

A total of 3296 infants were screened, and 928 (28%) were enrolled and vaccinated; 451/928 (49%) infants were chronically malnourished and randomized into MAL A and MAL B study arms; 477/928 (51%) were normally nourished and randomized into NOR A and NOR B study arms (Fig. 1). Both required samples of blood were received and analyzed from 852/928 (92%); both samples of stool were received and analyzed from 852/928 (92%) infants. All samples were received from 847/928 (91%) infants.

At enrolment the mean age was 10.4 months (IQR 9.6–11.2 months). Vaccination history was similar in all study arms with an average number of OPV doses received prior to enrollment being between 9 and 10. An average of 2 OPV doses were received as part of the routine immunization program; each child was exposed to an average of 8 additional OPV doses that were offered through SIAs conducted between birth of each child and enrolment. Between 2012 and 2013, the majority of SIAs in this area were with bOPV (80%) and the rest (20%) with tOPV. The SIAs in this area have been conducted on a monthly basis.

Diarrhea reported by parents that had occurred in the 7 days prior to enrollment was significantly more prevalent among the malnourished children (22%, 99/451) then among the normally nourished children (17%, 79/477) (*p* = 0.019).

**Fig. 2:**
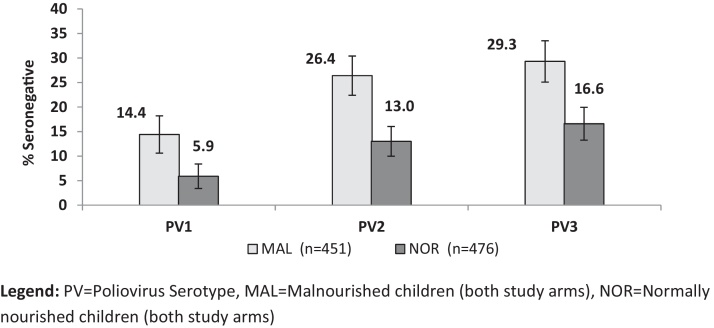
Proportion of immunologically unprotected children against poliovirus. PV = poliovirus serotype, MAL = malnourished children (both study arms), NOR = normally nourished children (both study arms).

For all three poliovirus serotypes, the baseline seroprevalence and median baseline titers were significantly lower among malnourished compared to normal infants (Table 2). At baseline, the seroprevalence was 85.6%, 73.6% and 70.3% in malnourished infants for poliovirus types 1, 2 and 3 respectively; compared with a seroprevalence of 94–1%, 87.0% and 83.4% among normal infants (Fig. 2). Among children who were seronegative for poliovirus serotype 1 at baseline, the seroconversion after one dose of bOPV alone was 19/35 (54.5%, CI95%: 36.6–71.2%) among malnourished and 4/8 (50%, CI95%: 15.7–84.3) among the normally nourished children. In comparison after one dose of IPV + bOPV the seroconversion rates were 17/22 (77.3%, CI95%: 54.6–92.2) and 15/16 (93.8%, CI95%: 69.8–99.8) for the malnourished and normally nourished infants, respectively. Among seronegative children for poliovirus serotype 2 at baseline, the seroconversion after one dose of IPV + bOPV was 41/49 (83.7%, CI95% 70.3–92.7) among the malnourished, and 24/24 (100%) among normally nourished children.

**Fig. 3:**
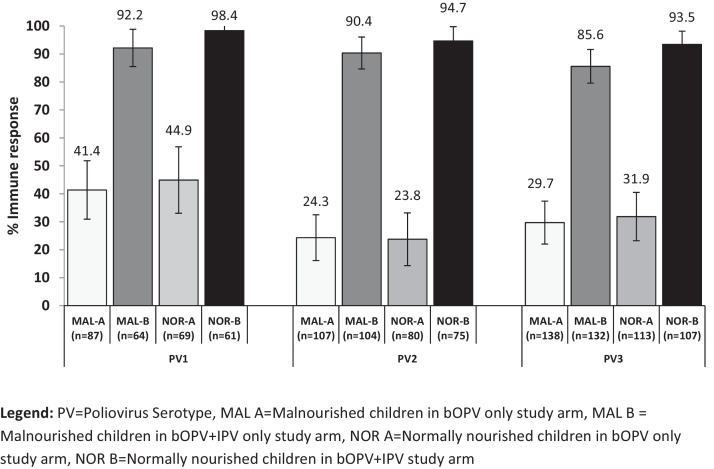
Immune response to one dose of bOPV or one dose of bOPV + IPV among malnourished and normally nourished children with baseline titer ≤362. PV = poliovirus serotype, MAL A = malnourished children in bOPV only study arm, MAL B = malnourished children in bOPV + IPV only study arm, NOR A = normally nourished children in bOPV only study arm, NOR B = normally nourished children in bOPV + IPV study arm.

The proportion of children who mounted an immune response (seroconversion or boosting) was significantly higher after one dose of IPV + OPV than after one dose of bOPV alone; and the proportion of children who mounted immune response among the malnourished was similar to that among normally nourished children for all three serotypes (Fig. 3).

**Fig. 4:**
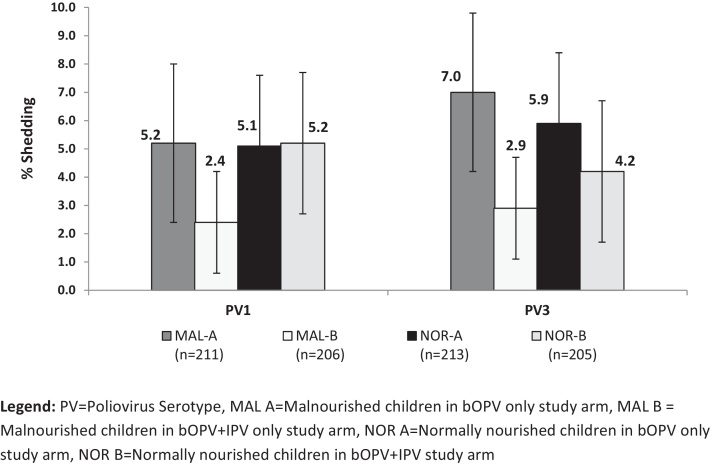
Proportion of vaccine poliovirus shedding 7 days after bOPV challenge. PV = poliovirus serotype; MAL A = malnourished children in bOPV only study arm; MAL B = malnourished children in bOPV + IPV only study arm; NOR A = normally nourished children in bOPV only study arm; NOR B = normally nourished children in bOPV + IPV study arm.

Shedding of vaccine polioviruses in stool was measured before and 7 days after a challenge dose with bOPV. Overall, the proportion of shedding was low (<10% in any study arm and for any poliovirus type). There were no significant differences detected in proportion of subjects shedding polioviruses between malnourished and normally nourished children (Fig. 4). Among the malnourished, there was an indication of reduction in shedding in the bOPV + IPV study arm compared with bOPV only arm (for PV1: *p* = 0.074, for PV 3: *p* = 0.036) (Fig. 4).

There were no severe adverse events causally linked to this study, however, there was one death reported in an infant who received bOPV only. This infant was admitted to a tertiary hospital with severe diarrhea, vomiting, and dehydration where he passed away within 24 h of admission. The symptoms occurred 21 days after bOPV administration. The Principal Investigator and the Data and Safety Monitoring Board did not attribute this death to the study procedures.

## 4 Discussion

At the start of this study, the chronically malnourished infants had lower seroprevalence against all three poliovirus serotypes than normally nourished infants despite having similar OPV vaccination history and age. The malnourished infants responded equally well to bOPV + IPV and to bOPV as the normal infants. Furthermore, a single dose of bOPV + IPV closed most of the remaining immunity gap against type 2 in both groups. In addition, the malnourished children reported more episodes of diarrhea occurring in the week prior to the enrollment. In Pakistan, chronic malnutrition is most likely a result of poor diet as well as poor environmental sanitation and hygiene leading to repeated episodes of intestinal infections and diarrhea [13,22,23]. Repeated episodes of intestinal infections and diarrhea lead to malnutrition and also contribute to lower OPV immunogenicity [10].

Our study population was well vaccinated with OPV: during 2011–2013 high quality OPV campaigns had been organized in and around Karachi almost every month reaching most children under 5 years of age. Therefore the subjects in the study had received an average of 10 OPV doses prior to enrollment; and yet there were 13% of malnourished children unprotected for poliovirus type 1. As previously observed, IPV in combination with OPV induced much superior immune response to OPV alone: less than half mounted immune response after one dose of bOPV alone, however, close to 100% responded to simultaneous IPV + bOPV dose.

This study had some limitations. The exact OPV vaccination history of the subjects was unknown. It was not possible to obtain a reliable estimate of OPV doses that children had received through SIAs. We assumed that the majority of children received most of the OPV doses offered through SIAs based on the data received from the Karachi polio program. Furthermore, the baseline titers of poliovirus neutralizing antibodies were high which may have impacted immune response to subsequent OPV doses. Our study did not address acutely malnourished infants because of ethical concerns. However, there are suggestions that acute malnutrition is a risk for lower immunogenicity in these children [9,10].

As the polio eradication program strives to reach its finish line it becomes increasingly important to ensure that everybody is protected against polioviruses. In our study, we observed that malnourished children are more likely to remain unprotected despite multiple doses of OPV compared to their normally nourished peers. One dose of bOPV + IPV administered to malnourished children raises their immunity level to parity with normally nourished children. Targeted bOPV + IPV campaigns in areas with known high rates of malnutrition and persistent wild poliovirus circulation are an opportunity to close the existing immunity gap. In Pakistan bOPV + IPV, when introduced in 2015, will be administered to all children reaching 14 weeks of age, however, older children will not benefit from the superior protection IPV offers, therefore we believe that additional bOPV + IPV campaigns, for example in the Federally Administered Tribal Areas (FATA), would significantly accelerate poliovirus eradication.

## Funding

The World Health Organization.

## Ethical approval

The study was approved by the Ethical Review Committee of the Aga Khan University, the National Bioethics Committee of Pakistan and the Ethical Review Committee of the World Health Organization, Geneva. All activities followed the guidelines of Good Clinical Practice; the trial protocol was registered at ClinicalTrials.gov with identifier NCT01695798. The World Health Organization assisted in study design, trial monitoring, and contributed to writing of the report. The Aga Khan University conducted the trial. Laboratory testing of stool was performed in the National Institute of Health, Islamabad, Pakistan; sera were tested at the Centers of Disease Control and Prevention, Atlanta, USA.

## Authors’ contribution

AFS and OM contributed equally to the manuscript. AF oversaw study implementation and data collection, wrote the first draft of the manuscript, was involved in data analysis and interpretation, and finalization of the manuscript. FQ implemented the study at the field sites, supervised data collection, wrote the first draft of Section 2 and was involved in data analysis and interpretation. OM was involved in study design, trial monitoring, and data analysis plan and data interpretation. He was also involved in finalization of the manuscript. ZB was responsible for data analysis. AK was responsible for quality control in field sites during study procedures and data collection. NR was responsible for data analysis. MS was responsible for trial monitoring and compliance with Good Clinical Practice (GCP) guidelines. SMO was responsible for neutralization antibody assays and finalization of the manuscript. WCW was responsible for neutralization antibody assays and finalization of manuscript. RWS was the senior adviser on study design and implementation, and, data interpretation. AKMZ was the principal investigator for the study and the overall guarantor; she oversaw study design, implementation, analysis and the manuscript writing. All authors reviewed and approved the final manuscript.

## Figures and Tables

**Table 1:** Demographic characteristics of study participants.

	Malnourished Mal A (bOPV)	Malnourished Mal B (bOPV + IPV)	Normally nourished Nor A (bOPV)	Normally nourished Nor B (bOPV + IPV)	*p*-value
	(*n* = 224)	(*n* = 227)	(*n* = 239)	(*n* = 238)	
Gender					
Female	107 (47.8)	111 (48.9)	120 (50.2)	134 (56.3)	NS
Age (months)	10.5	10.5	10.4	10.3	NS
Anthropometry of the study population					
WHZ score ± SD	−0.92 ± 0.87	−0.93 ± 0.87	−0.37 ± 0.95	−0.45 ± 0.85	
HAZ score ± SD	−2.92 ± 0.76	−2.86 ± 0.74	−0.93 ± 0.85	−0.94 ± 0.84	
WAZ score ± SD	−2.33 ± 0.81	−2.30 ± 0.82	−0.76 ± 0.90	−0.84 ± 0.86	
Vaccination history					
EPI fully immunized children: 4 OPV doses received *n* (%)	104 (46.4)	124 (54.6)	129 (54)	141 (59.2)	NS
EPI OPV Doses (Average Received: tOPV)	2.4	2.7	2.7	2.8	NS
SIA OPV Doses (average exposure: bOPV or tOPV)	8.4	8.4	7.1	7.1	NS
Self-reported diarrhea					
Diarrhea within 7 days prior to enrollment	48 (21.4)	51 (22.5)	41 (17.2)	38 (16)	*p* = 0.019

**Table 2:** Baseline and 28 days post vaccination seroprevalence and median titers.

Baseline		MAL-A bOPV Only	MAL-B bOPV + IPV	NOR-A bOPV Only	NOR-B bOPV + IPV	*p* value
		(*n* = 224)	(*n* = 227)	(*n* = 239)	(*n* = 238)	
**PV1**	Seropositive *n* (%)	187 (83.5)	199 (87.7)	230 (96.2)	219 (92)	<0.001
	Median titer (CI)	724.1 (455.1, 929.4)	910.2 (724.1, 1152.1)	1152.1 (910, 1448.2)	1448.2 (1152.1, 1448.2)	0.002

**PV2**	Seropositive *n* (%)	160 (71.4)	172 (75.8)	204 (85.4)	211 (88.7)	<0.001
	Median titer (CI)	408.6 (219.7, 755.6)	362 (191.6, 576)	910.2 (576, 1152.1)	910.2 (724.1, 1152.1)	<0.001

**PV3**	Seropositive *n* (%)	159 (71)	160 (70.5)	200 (83.7)	198 (83.2)	<0.001
	Median titer (CI)	181 (56.9, 288)	72 (45.3, 181)	362 (227.5, 455.1)	362 (227.5, 576)	<0.001

28 Days post vaccination		(*n* = 213)	(*n* = 206)	(*n* = 216)	(*n* = 212)	*p* value
**PV1**	Seropositive *n* (%)	195 (91.5)	201 (97.6)	212 (98.1)	211 (99.5)	<0.001
	Median titer (CI)	1448.2 (1075.9, 1448.2)	1448.2 (1448.2, 1448.2)	1448.2 (1448.2, 1448.2)	1448.2 (1448.2, 1448.2)	>0.05

**PV2**	Seropositive *n* (%)	163 (76.5)	198 (96.1)	185 (85.6)	211 (99.5)	<0.001
	Median titer (CI)	576 (288, 724.1)	1448.2 (1448.2, 1448.2)	1152.1 (910.2, 1448.2)	1448.2 (1448.2, 1448.2)	>0.05

**PV3**	Seropositive *n* (%)	160 (75.1)	189 (91.7)	185 (85.6)	207 (97.6)	<0.001
	Median titer (CI)	227.5 (134.5, 362)	1448.2 (1448.2, 1448.2)	455.1 (288, 724.1)	1448.2 (1448.2, 1448.2)	>0.05
