# Supplementary material for: Immunogenicity of poliovirus vaccines in chronically malnourished infants: A randomized controlled trial in Pakistan
Source: Vaccine. 2015 Jun 4;33(24):2757–63. doi: 10.1016/j.vaccine.2015.04.055 (PMC4447616; doi:10.1016/j.vaccine.2015.04.055)
Supplement: Supplementary file 1 [file mmc1.doc]

| Figure.  Geographical land marks of research Sites, Department of Paediatrics and Child Health, Aga Khan University Karachi, Pakistan |
| --- |
| 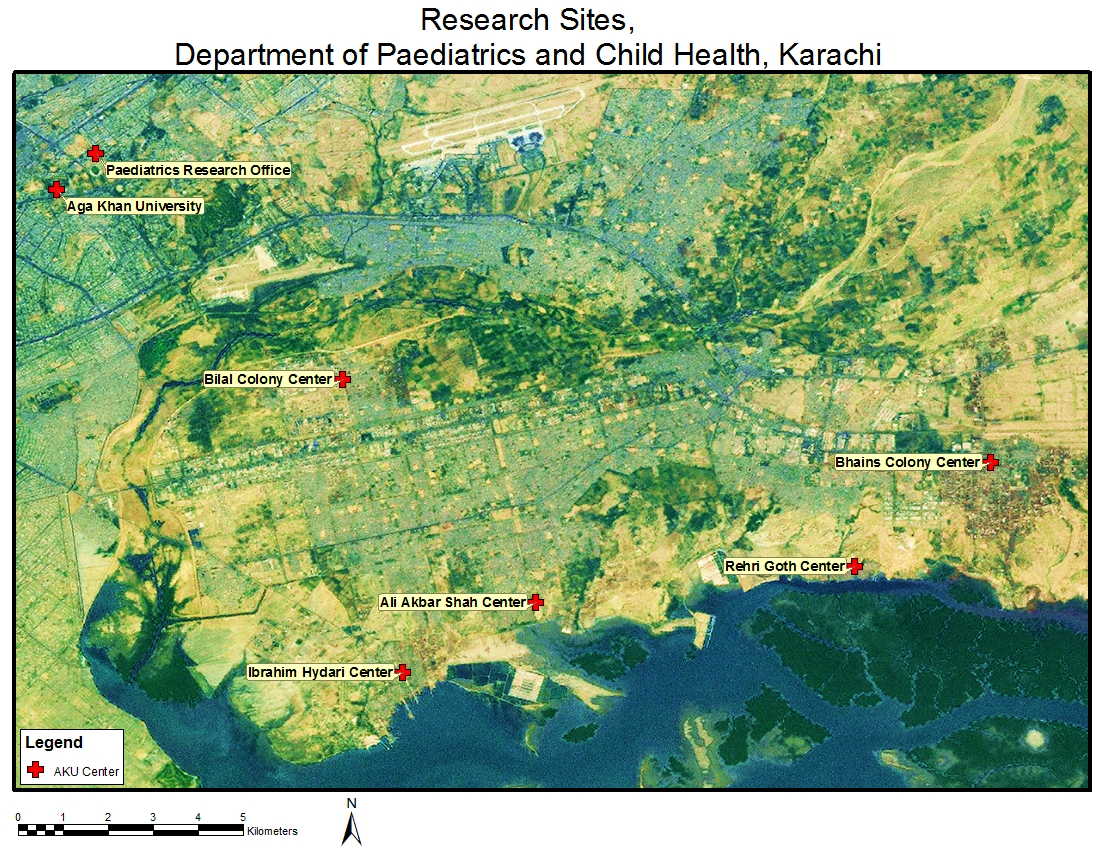 |
| **+**, Primary health center and Research Sites, Department of Paediatrics and Child Health, Aga Khan University, Karachi, Pakistan  , (Diamond), indicate the sites where study was conducted |
